# Is Routine Ultrasound Examination of the Gallbladder Justified in Critical Care Patients?

**DOI:** 10.1155/2012/565617

**Published:** 2012-05-09

**Authors:** Pavlos Myrianthefs, Efimia Evodia, Ioanna Vlachou, Glykeria Petrocheilou, Alexandra Gavala, Maria Pappa, George Baltopoulos, Dimitrios Karakitsos

**Affiliations:** ^1^Department of Intensive Care at “Agioi Anargyroi” General Hospital, Faculty of Nursing, University of Athens, Kaliftaki, 14564 Nea Kifissia, Greece; ^2^Department of Intensive Care Unit, General State Hospital of Athens, Athens, Greece

## Abstract

*Objective*. We evaluated whether routine ultrasound examination may illustrate gallbladder abnormalities, including acute acalculous cholecystitis (AAC) in the intensive care unit (ICU). *Patients and Methods*. Ultrasound monitoring of the GB was performed by two blinded radiologists in mechanically ventilated patients irrespective of clinical and laboratory findings. We evaluated major (gallbladder wall thickening and edema, sonographic Murphy's sign, pericholecystic fluid) and minor (gallbladder distention and sludge) ultrasound criteria. *Measurements and Results*. We included 53 patients (42 males; mean age 57.6 ± 2.8 years; APACHE II score 21.3 ± 0.9; mean ICU stay 35.9 ± 4.8 days). Twenty-five patients (47.2%) exhibited at least one abnormal imaging finding, while only six out of them had hepatic dysfunction. No correlation existed between liver biochemistry and ultrasound results in the total population. Three male patients (5.7%), on the grounds of unexplained sepsis, were diagnosed with AAC as incited by ultrasound, and surgical intervention was lifesaving. Patients who exhibited ≥2 ultrasound findings (30.2%) were managed successfully under the guidance of evolving ultrasound, clinical, and laboratory findings. *Conclusions*. Ultrasound gallbladder monitoring guided lifesaving surgical treatment in 3 cases of AAC; however, its routine application is questionable and still entails high levels of clinical suspicion.

## 1. Introduction

Abnormalities of the gallbladder (GB) are frequent in the intensive care unit (ICU) [1, 2]. Critical care patients have many risk factors for acute acalculous cholecystitis (AAC) which is an acute inflammation of the GB in the absence of gallstones and accounts for 2–14% of all cases of acute cholecystitis [3–5]. AAC is an insidious complication that has been increasingly recognized in the critically ill with an incidence ranging from 0.2 to 3% [6–8].

Although the etiology is uncertain, AAC in the ICU has been associated with prolonged enteral fasting, total parenteral nutrition (TPN), duration of mechanical ventilation (MV) and the use of positive end-expiratory pressure (PEEP), activation of factor XII, trauma, sepsis, drugs (opiates, sedatives, and vasopressors), multiple transfusions, dehydration, and shock states [6, 9, 10]. Ultimately these factors may lead to bile stasis and GB hypoperfusion/ischemia resulting in acute inflammation of the GB.

AAC is an emergency condition, and without immediate treatment there may be rapid progression to gangrenous cholecystitis (approximately 50%) or perforation (approximately 10%), with mortality rates as high as 65% [11]. However, with timely diagnosis and intervention, the mortality drops to 7%. The clinical presentation is variable and often depends upon the underlying predisposing conditions. 

Hence, we evaluated whether the routine application of standardized GB sonographic examination upon critical care patients, irrespective of their clinical presentation and/or laboratory findings, might aid in identifying the range and the significance of GB abnormalities, including AAC, and consequently affect our decision making.

## 2. Patients and Methods

### 2.1. Study Cohort

This prospective study was conducted in a seven-bed ICU based at KAT General Hospital, Athens, Greece, following approval from our institutional ethics committee. All patients who were admitted to the ICU during an 8-month period (1/5/2011–1/12/2011) were enrolled in this study. Right-upper quardant sonography was performed by means of a Vivid 4 portable ultrasound system (GE, Medical System, Waukesha, WI, USA) equipped with a convex 5 to 7 MHz transducer. Sonographic examinations were performed upon admission, while follow-up examinations were performed twice weekly until patients were either discharged from the ICU or expired, in all cases. Upon admission, all patients were sedated using midazolam or propofol and/or remifentanil according to recommended doses and clinical response; moreover, they were mechanically ventilated (Taema HORUS Ventilator, Air Liquide, *Paris Cedex, *France). All sonographic examinations were performed by two independent and experienced radiologists who were blinded to patients' identity.

### 2.2. Definitions and Outcomes

Abdominal sonographic investigations were focused on the GB as previously described [1–4, 9]. AAC was defined as acute inflammation of the gallbladder in the absence of gallstones [12]. Ultrasound findings that were evaluated included major criteria: gallbladder wall thickening (greater than 3 mm), striated (edematous) gallbladder wall, sonographic Murphy's sign, pericholecystic fluid in the absence of ascites, and hypoalbuminemia and minor criteria: gallbladder distention (hydrops with a long-axis caliper over 100 mm and a short axis (transverse diameter) over 50 mm) and biliary or gallbladder sludge [1, 2, 9, 13, 14]. Hepatic dysfunction was defined as bilirubin >2 mg/dL and/or alkaline phosphatase (ALP) >200 IU/L [15]. Pertinent clinical and laboratory parameters were recorded: demographics, temperature, WBC, MV status, liver function tests, and administration of parenteral nutrition, narcotic analgesics, and vasopressor agents, and predisposing factors which are associated with high incidence AAC.

### 2.3. Statistics

Continuous data are presented as means ± SD. Categorical data are presented as numbers and percentages. Relationships between categorical variables were tested with chi-square analysis. Tests were two sided, and *P* < 0.05 was considered statistically significant. All data were analyzed using SPSS 17.0 software (SPSS Inc. Chicago, IL, USA).

## 3. Results

Fifty-three consecutive critical care patients participated in this study. Demographics, admission diagnosis, severity of illness, and mortality rate of the study population are presented on Table 1.

There were 1680 days of ICU hospitalization and 265 gallbladder/biliary tract ultrasound examinations (median 5.1, mean ± SEM 4.7 ± 2.1 per patient) recorded. Twenty-five patients (47.2%) exhibited at least one abnormal GB finding on ultrasound examination, while 16 patients (30.2%) had two or more concomitant findings. Imaging findings are presented in Table 2. Of the 25 patients who exhibited at least one sonographic finding, only six patients (24%) presented concomitant hepatic dysfunction, while 3 patients (12%) had solely increased **γ**-glutamyltransferase (*γ*-GT ≥ 150 IU/Lt, 415.3 ± 50.2) and 2 patients (8%) had solely increased alanine transaminase (ALT ≥ 150 IU/Lt, 217.5 ± 31.2), respectively. Hence, patients with at least one positive imaging finding and normal liver biochemistry results were significantly more than patients with hepatic dysfunction (**χ*^2^*, *P* = 0.0005). In contrast, 23 (82.1%) out of the 28 patients with normal ultrasound findings exhibited transient abnormalities in liver function tests but no hepatic dysfunction. These included increased transaminases, bilirubin, ALP, or **γ**-GT which were after meticulous investigation attributed to reasons other than AAC (drugs, sepsis, trauma-rhabdomyolysis, etc.).

**Figure 1:**
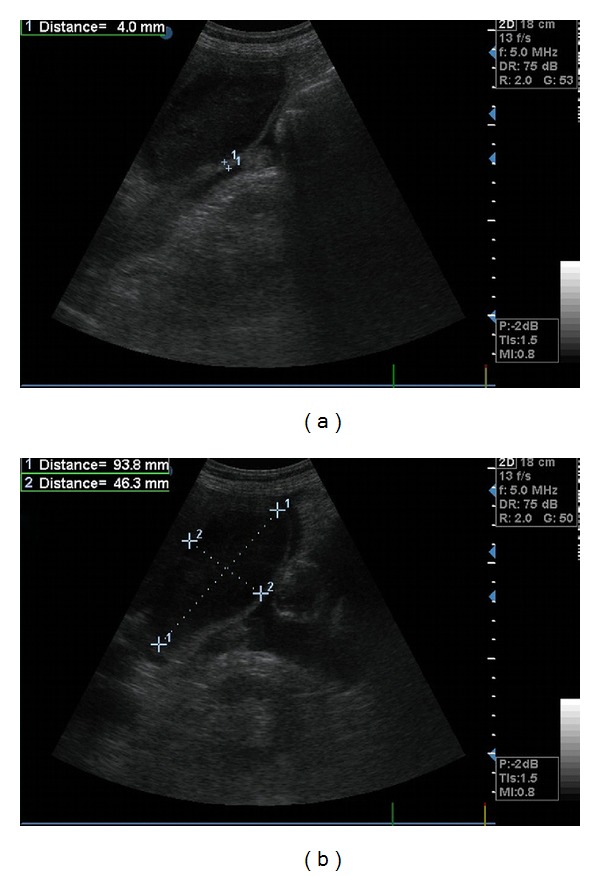
Gallbladder ultrasound depicting one patient with acute acalculous cholecystitis exhibiting wall thickening (4 mm) in the presence of sludge (a) and marginally increased dimensions (93 × 46.3 mm) with pericholecystic fluid (b).

Notably, 3 male trauma victims (5.7%), during the course of their hospitalization, presented with clinical features of sepsis without definite source of infection (unexplained fever, leucocytosis, hemodynamic instability). The above patients had sonographic findings compatible with AAC and consequently underwent urgent open cholecystectomy as decided by the attending intensivist and the surgeon in charge (Figure 1). All 3 patients exhibited sonographic findings of gallbladder wall thickening (>3.5 mm), marginally increased GB dimensions, and pericholecystic fluid. All of them were under MV, vasopressors, midazolam and remifentaniyl, and TPN. Only one out of these three patients with AAC showed evidence of hepatic dysfunction as defined [15]. Day of surgery was the 14th, 22nd, and 42nd of ICU stay, respectively, leading to clinical improvement of the patients that is apyrexia and gradual discontinuation of vasopressors.

The 13 (24.5%) patients exhibiting ≥2 imaging findings but not AAC were managed successfully by applying measures including gastric drainage and modulation of antibiotic therapy to cover possible pathogens originating from the gallbladder and/or interruption of enteral or parenteral nutrition, under the guidance of evolving ultrasound, clinical, and laboratory findings. None of these patients exhibited AAC, while hepatic dysfunction was present only in 2 cases (15.4%).

Finally, 19 patients (35.8%, 14 with US findings) did not have any liver function tests abnormalities and 34 patients (64.2%) had liver function tests abnormalities, of whom only 11 (32.4%) had concomitant US findings. Only 5 (9.4%) patients had both normal liver function and normal ultrasound findings of the GB during their ICU stay.

## 4. Discussion

AAC poses major diagnostic challenges in critical care patients.GB abnormalities and AAC are one of the many potential causes in the differential diagnosis of systemic inflammatory response syndrome and sepsis or jaundice and no other obvious source of infection [11]. Notably, gallbladder ischemia can progress rapidly to gangrene and perforation with detrimental effects. Indeed physical examination and laboratory evaluation are unreliable in AAC [16]. Abdominal pain and tenderness may be masked by analgesia and sedation. Fever is generally, present but other physical findings may not be consistent and/or reliable, particularly physical examination of the abdomen [17]. Leukocytosis and jaundice are commonplace, but nonspecific in the setting of critical illness. Also, a number of pitfalls can be encountered in the interpretation of common liver function tests [18, 19]. Alterations of hepatic enzymes reflecting the extent of hepatocellular necrosis (i.e., transaminases) or cholestasis (i.e., bilirubin) could be attributed to various causes such as extrahepatic infection and sepsis, ischemia/reperfusion injury, total parenteral nutrition, trauma, and drug adverse effects. Diagnosis of intra-abdominal pathology and AAC often rests on imaging studies and clinical suspicion [11]. Computed tomography scans are useful but can be ambiguous, while oftentimes the patient is too unstable to be safely transferred. Ultrasound by-the-bed examination represents not only an alternative imaging method, but also a lifesaving diagnostic tool in the detection of intra-abdominal pathology and remains the screening procedure of choice for depicting GB abnormalities [15–22].

In this study, almost half of our patients (47.2%) exhibited at least one GB abnormality on ultrasound examination and 30.2% of them had ≥2 findings. In fact, anomalies of GB are extremely common and could be found in up to 84% of the critically ill as a result of various causes [1, 2, 23]. However, we found that only 5.7% of the patients developed AAC requiring surgical intervention which is higher than that reported in the literature [6–8]. In a previous study, 14 out of 28 critical care patients (50%; 19 intubated) were found to have one of the three major sonographic criteria for AAC, but none of these subjects needed any intervention [23]. It was suggested that thickening of the gallbladder wall is the single most reliable criterion, with reported specificity of 90% at 3 mm and 98.5% at 3.5 mm wall thickness and sensitivity of 100% at 3 mm and 80% at 3.5 mm [24–26]. Accordingly, gallbladder wall thickness greater than or equal to 3.5 mm is generally accepted to be diagnostic of AAC [24–26]. In our cohort, 19 patients had gallbladder wall thickening >3 mm, but only 3 developed AAC compatible with the clinical condition of the patients. Other helpful sonographic findings for AAC such as pericholecystic fluid, striated gallbladder wall, and distention of the gallbladder of more than 5 cm were found in five, three and eight patients, respectively. In this study, all patients who exhibited AAC presented with GB wall thickening >3.5 mm and pericholecystic fluid; however, the recorded rate of AAC was too low to justify routine ultrasound examination of the GB on a weekly basis. The present findings suggest that on the grounds of clinical suspicion for AAC (i.e., unexplained sepsis syndrome), even in the absence of liver dysfunction, a sonographic examination could alter the decision making and could be potentially lifesaving for the individual patient. 

Furthermore, 13 (24.5%) of our patients who presented with ≥2 ultrasound findings, of whom only 2 had liver dysfunction, were medically managed (gastric drainage, antibiotics, interruption of enteral nutrition, etc.) under the guidance of evolving ultrasound, clinical, and laboratory parameters. 

Nevertheless, alterations in liver function tests were not correlated to pertinent ultrasound findings in this cohort of critical care patients. It is worth mentioning that *≈*64% (34/53) of our patients had liver dysfunction of which only 32% (11/34) had concomitant gallbladder US findings. That is, for 23 patients having liver dysfunction, US examination was crucial to exclude GB abnormalities. On the contrary, in the 3 patients with AAC only one presented with concomitant hepatic dysfunction. Surely, routine evaluation of liver function tests for diagnosing AAC is neither specific nor sensitive. AAC represents an underdiagnosed entity in the ICU, and this may be partially due to the complexity of underlying medical and surgical problems and lack of reproducible signs and biochemical parameters [1, 6, 9]. Diagnosis of AAC and GB anomalies in general relies on a high level of clinical suspicion. Moreover, AAC is considered an ischemic rather than an infectious disorder, and any abdominal pain in a critically ill patient, or even unexplained fever or hemodynamic instability, warrants consideration of this diagnosis [2]. Prompt application of ultrasound investigations could confirm clinical suspicions and guide consequently therapeutic options [1, 9, 27, 28]. 

### 4.1. Limitations

This study has many limitations. Ultrasound is a method with inherent technical limitations; moreover increased body mass index, subcutaneous edema, and/or air and mechanical ventilation may affect the clarity of ultrasound images in the ICU [23, 24, 28]. Also, the sample of patients was rather small to perform any meaningful subgroup analysis and to draw definite conclusions about the correlation of laboratory and imaging findings in patients with GB anomalies. Future larger prospective studies are required to investigate further the issues raised in this study. Despite the aforementioned limitations, ultrasound is a useful diagnostic tool for detection of AAC and GB abnormalities in the ICU. Its prompt application may aid in altering therapeutic strategies, operative or conservative, and could prove lifesaving for the individual patient.

### 4.2. Conclusions

In this study, alterations in liver function tests were not correlated to pertinent ultrasound findings in critical care patients with GB abnormalities. Standardized ultrasound monitoring of the GB facilitated the diagnosis of 3 cases of AAC and thus guided prompt surgical treatment. The former accordingly guided the medical management of 13 patients who exhibited two or more imaging findings without ACC and excluded GB abnormalities in 23 patients having abnormal liver function test alone. However, the low rate of AAC observed in this small series could not justify routine ultrasound examination of the GB to identify AAC in the ICU. On the other hand routine ICU ultrasound examination was found useful in almost 75% of ICU patients for differential diagnosis, monitoring of abnormalities found or therapies applied, or excluding GB abnormalities. Taking into account this high rate in combination with the bedside availability of US examination, the capability to investigate other organs such as heart, vessels, and lungs, and the low related costs, ultrasound examination can be an examination of choice in most critically ill ICU patients.

## Figures and Tables

**Table 1:** Clinical characteristics of the study population.

Total number of patients	*n* = 53
Age (years)	57.6 ± 2.8
Male gender (%)	42 (79.2%)
Admission diagnosis	Trauma-burns: 27 (50.9%) postsurgical complications: 8 (15.1%) SAH: 7 (13.2%) medical: 11 (20.7%)
APACHE II (mean ± SD)	21.3 ± 0.9
SAPS II (mean ± SD)	53.3 ± 2.3
SOFA score (mean ± SD)	10.2 ± 0.2
ICU stay (days) (mean ± SD)	35.9 ± 4.8
Mortality	17/53 (32.1%)

Abbreviations are: SAH: acute subarachnoid hemorrhage; APACHE: acute physiology and chronic health evaluation score; SAPS: simplified acute physiology score; SOFA: sequential organ failure assessment; ICU: intensive care unit.

**Table 2:** Ultrasound results in the 25 patients who exhibited at least one finding.

Total number of patients with at least one finding	25/53 (47.2%)
Gallbladder wall thickening (>3 mm)	19/25 (76%)
Gallbladder distention (long axis > 100 mm, short axis > 50 mm)	8/25 (32%)
Striated gallbladder wall	3/25 (12%)
Pericholecystic fluid	5/25 (20%)
Gallbladder sludge	19/25 (76%)
